# Xanthohumol induces paraptosis of leukemia cells through p38 mitogen activated protein kinase signaling pathway

**DOI:** 10.18632/oncotarget.16185

**Published:** 2017-03-14

**Authors:** Xiangquan Mi, Chunming Wang, Chao Sun, Xu Chen, Xiang Huo, Yiming Zhang, Gang Li, Bo Xu, Jun Zhang, Jianxin Xie, Zhenhua Wang, Ji Li

**Affiliations:** ^1^ School of Life Sciences, Lanzhou University, Lanzhou, Gansu 730000, P.R. China; ^2^ Center for Mitochondrial and Healthy Aging, College of Life Sciences, Yantai University, Yantai, Shandong 264005, P.R. China; ^3^ Institute of Modern Physics, Chinese Academy of Sciences, Lanzhou 730000, P.R. China; ^4^ Shihezi University School of Medicine, Shihezi, Xinjiang 832000, P.R. China

**Keywords:** xanthohumol, paraptosis, p38-MAPK, cytoplasmic vacuolation, ER stress

## Abstract

Xanthohumol as a natural polyphenol demonstrates an anticancer activity, but its underlying mechanism remains unclear. In this study, we showed that xanthohumol (XN) induces paraptosis of leukemia cells. The paraptosis is one cell death which is characterized by dilation of the endoplasmic reticulum and/or mitochondria. The results demonstrated that XN treatment significantly inhibited cell proliferation and triggered extensive cytoplasmic vacuolation of HL-60 leukemia cells, but it did not cause the cleavage of caspase-3 protein or apoptosis. In contrast, XN treatment resulted in LC3-II accumulation through blocking of autophagosome maturation. Interestingly, the induction of cytoplasmic vacuolization by XN is not associated with autophagy modulated by XN, therefore, XN-induced cell death of HL-60 leukemia cells is not the classical apoptotic cell death. Intriguingly, XN treatment triggered the dilatation of endoplasma reticulum (ER) and induced ER stress by upregulating C/EBP homologous protein and unfolded protein response regulator Grp78/Bip. Furthermore, XN treatment triggered p38 mitogen activated protein kinase and its specific inhibitor inhibited the paraptosis of HL-60 leukemia cells by XN. In conclusion, we for the first time demonstrated that XN treatment can induce paraptosis of leukemia cells through activation of p38 MAPK signaling.

## INTRODUCTION

The chemotherapeutic agents mostly demonstrate anti-cancer activity through caspase-dependent apoptosis [1]. The chemotherapy resistance for cancer cells is a limited factor for the success of antitumor drugs in cancer treatment. These could be due to genetic mutations in the pro-apoptotic protein of Bax and augment of anti-apoptotic proteins of Bcl-2 and X-chromosome linked inhibitor of apoptosis [2, 3]. Therefore, the therapies according to triggering non-apoptotic cell death could provide a good approach for treatment of cancers that are resistant to multi-drugs-induced apoptosis [4].

Paraptosis is characterized by cytoplasmic vacuolation that could be generated from swelling of the endoplasmic reticulum and/or mitochondria. The paraptosis usually does not have caspase activation an apoptotic hallmark [5, 6]. Moreover, there is evidence that a protein synthesis inhibitor cycloheximide (CHX) can block the formation of cytoplasmic vacuole in cytoplasmic vacuolation-mediated cell death and paraptosis [6, 7]. A previous report showed that autophagic marker LC3-II are upregulated and the endoplasmic reticulum stress markers unfolded protein response regulator Grp78/Bip and transcription factor CHOP are observed in paraptosis of cancer cells [5]. In contrast, regarding whether a paraptosis process is involved in cell death of leukemia cells and dopaminergic neuroblastoma cells have not been elucidated [8]. Several natural products such as curcumin, celastrol, ophiobolin A and paclitaxel have demonstrated anti-cancer activities through the induction of paraptosis-related cell death, but there is no study to show the direct antitumor effects of Xanthohumol (XN) via paraptosis.

**Figure 1:**
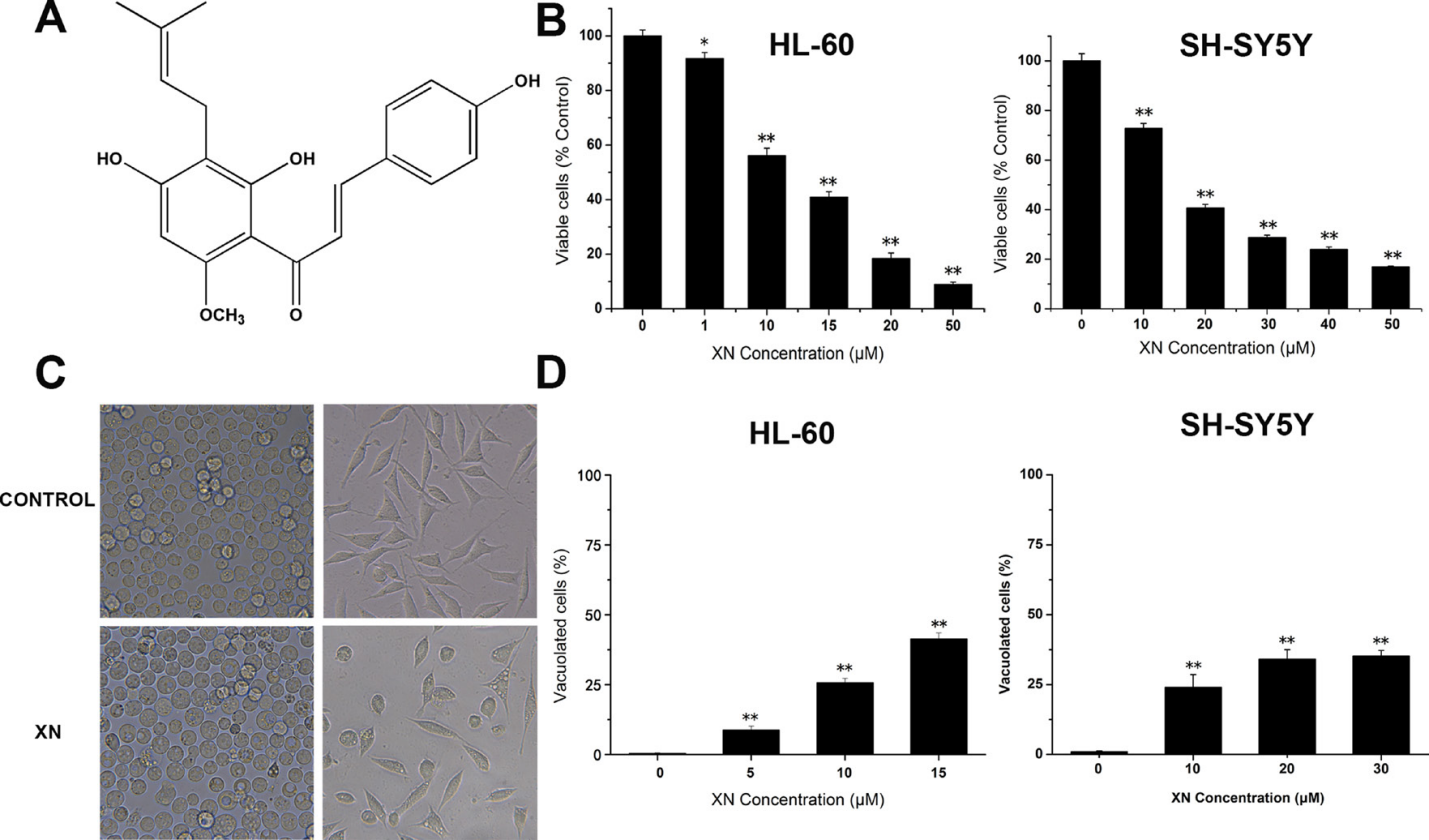
XN treatment results in reduced cell viability and morphological changes in cancer cells (**A**) The chemical structure of xanthohumol (XN). (**B**) HL-60 leukemia cells and SH-SY5Y neuroblastoma cells were treated for 48 h with various concentrations of XN (1–50 μM). The cell viability of HL-60 cells was measured by trypan blue staining and the cell viability of SH-SY5Y cells was assessed using CCK-8 assay. (**C**) The cells were treated with XN at 15 μM for 48 h, and morphology was examined using light microscope. (**D**) The cells were treated with different concentrations of XN for 48 h, and the percentage of vacuolated cells was measured using light microscope. All data are presented as mean ± S.D. from three independent experiments. *P < 0.05, **P < 0.01 *vs*. control.

Hops (*Humulus lupulus L*.) flowers have been used as a raw material in the industry to generate aroma and flavor. Besides the application of hops in the industry, they have been also used for different medical purposes. Xanthohumol is an important flavonoid in hops. Xanthohumol (XN) (Figure 1A) is a prenylated chalcone that was isolated from the female hop plant, *Humulus lupulus*, possesses proven pharmacologic safety and multiple bioactivities, including anti-cancer, anti-diabetes, anti-inflammatory and anti-bacteria [9]. In current study, we demonstrated that XN induces paraptosis of HL-60 leukemia cells, and the paraptosis occurred in HL-60 cells is characterized with cytoplasmic vacuolation and dilated endoplasmic reticulum triggering endoplasmic reticulum (ER) stress. In addition, our results revealed that p38 MAPK plays a critical role in the XN-induced paraptosis. Therefore, the paraptosis of leukemia cells induced by Xanthohumol could be an implication of Xanthohumol for cancer therapeutics.

## RESULTS

### XN inhibits proliferation and induces cytoplasmic vacuolation

In order to investigate the anti-malignant tumor effects of XN, HL-60 leukemia cells were treated with Xanthohumol (approximately 0–50 μM) for 48 hours and cell viability assays were performed using Trypan blue assay; besides leukemia cells, the SH-SY5Y neuroblastoma cells were also tested the response to XN treatment, SH-SY5Y neuroblastoma cells were administrated with XN for 48 h and cell viability assays were performed by CCK8 assay, respectively. It was observed that XN dose-dependently decreased cell viability of both HL-60 leukemia cells and SH-SY5Y neuroblastoma cells (Figure 1B), suggesting a general cytotoxic effect of XN on cancer cells. Microscopic observation revealed that stimulation with higher XN concentrations (more than 50 μM) led to cell detachment. Treatment with XN induced extensive cytoplasmic vacuolation in HL-60 leukemia cells and SH-SY5Y neuroblastoma cells (Figure 1C), and the number of vacuolated cells was dose-dependently increased (Figure 1D). Compared with the SH-SY5Y neuroblastoma cells, the HL-60 leukemia cells displayed more sensitive to XN treatment, therefore, HL-60 leukemia cells are used for further determination of mechanisms by which XN induces paratosis of cancer cells.

### Apoptosis and autophagy are not involved in XN-induced cell death

**Figure 2:**
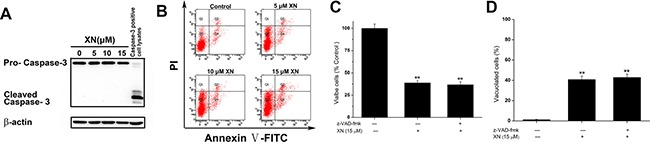
Apoptosis is not involved in the XN-induced cell death (**A**) The HL-60 leukemia cells were treated with 5, 10, 15 μM XN for 48 h, and caspase-3 protein levels were measured. (**B**) The cells were analyzed for effects of XN on apoptosis by Annexin V-FITC/PI staining, and fluorescence was analyzed using flow cytometry. (**C** and **D**) HL-60 cells were pretreated with z-VAD-fmk at 50 μM for 1 h before treatment of XN at 15 μM for 48 h. The percentage of viable cells (C) and vacuolated cells (D) were also measured. All data are expressed as mean ± S.D. from three independent experiments. **P < 0.01 *vs*. control group.

**Figure 3:**
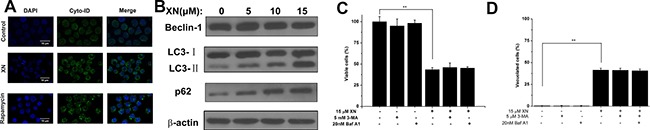
XN treatment alters autophagic influx (**A**) The HL-60 leukemia cells were treated with 15 μM XN for 48 h. The cells were then loaded with Cyto-ID for 30 min and immediately imaged by confocal microscopy. The cells were treated with 200 nM rapamycin overnight as positive control of autophagy. (**B**) The cells were treated with 5, 10, 15 μM XN for 48 h and immunoblotting was used to determine protein levels of Beclin- 1,LC3-II and p62. b-actin was used as a loading control. (**C** and **D**) The cells were treated with 3-MA (5 mM, 1h) and bafilomycin- A1 (20 nM, 1h) prior to treatment of XN at 15 μM for 48 h. The percentages of viable cells (C) and vacuolated cells (D) were measured. Data are expressed as mean ± S.D. from three independent experiments. **P < 0.01 *vs*. control group.

The results demonstrated that XN treatment induced cell death accompanying with cytoplasmic vacuolation that could a new type of cell death caused by XN. As in most literature reported, cell death induced by XN is attributed to apoptosis [10, 11]. Firstly, in order to examine caspase activation, HL-60 leukemia cells were administrated with 5 μM, 10 μM, and 15 μM of XN for 48 h, respectively, and the levels of caspase-3 were determined. When the cells were treated with XN, the full length of caspase-3 were not altered, and the cleaved fragments of caspase-3 were not identified (Figure 2A). Next, to further prove XN-treated cells did not induce apoptosis, we carried out flow cytometry analysis showed that XN did not significantly augmented the number of apoptotic cells (Figure 2B). The HL-60 leukemia cells were administrated with a caspase inhibitor z-VAD-fmk, prior to the treatment of XN, the number of viable cells and the cells with vacuolation (Figure 2C and 2D) was not changed obviously. Taken together, these results indicated that apoptosis might not contribute to the cytotoxicity of XN toward HL-60 cells. However, the HL-60 cells with XN treatment caused extensive cytoplasmic vacuolation, therefore, we further assessed whether XN-induced vacuolation and cell death were related to autophagic cell death. Firstly, we measured autophagy induction with the fluorescence microscopy and using Cyto-ID^®^ Green as a specific autophagy dye that does not stains lysosomes but can stain autophagosomes in the HL-60 leukemia cells, emitting green flurorescence and forming punctate structures. It clearly showed that there was no Cyto-ID fluorescence was detected in the control group, but HL-60 leukemia cells treated with 15 μM of XN for 48 h increased the level of Cyto-ID signal (green fluorescence) (Figure 3A). The increase in green fluorescence could be related with phosphatidylethanolamine (PE) conjugation. The augmented formation of autophagosomes could be a reason, another reason is the impaired maturation of autophagosomes-induced block of LC3-II degradation. In order to distinguish the difference of the two possibilities, we tested the protein expression levels of Beclin-1, LC3-II and p62. There is evidence that Beclin-1 as a critical protein mediating the activation of autophagy and the Beclin-1 has a short BH3 motif to interact with Bcl-2. When the HL-60 leukemia cells were administrated with 5 μM, 10 μM and 15 μM of XN for 48 h, respectively, the increased expression levels of LC3-II and p62 but not Beclin-1 were found (Figure 3B). These results suggested that XN treatment-induced the up-regulated LC3-II expression could be an attenuation of autophagosome maturation. However, we found that the HL-60 leukemia cells were pre-administrated with the inhibitors of autophagy, 3-MA or bafilomycin A1 did not have any effects on XN induced cell death (Figure 3C) and XN-induced cellular vacuolation (Figure 3D). Taken together, these results strongly indicated that the apoptotic or autophagic cell death pathway is not involved in the XN-induced cytoplasmic vacuolation mediated HL-60 leukemia cell death.

### Paratosis is occurred with XN treatment of HL-60 leukemia cell

**Figure 4:**
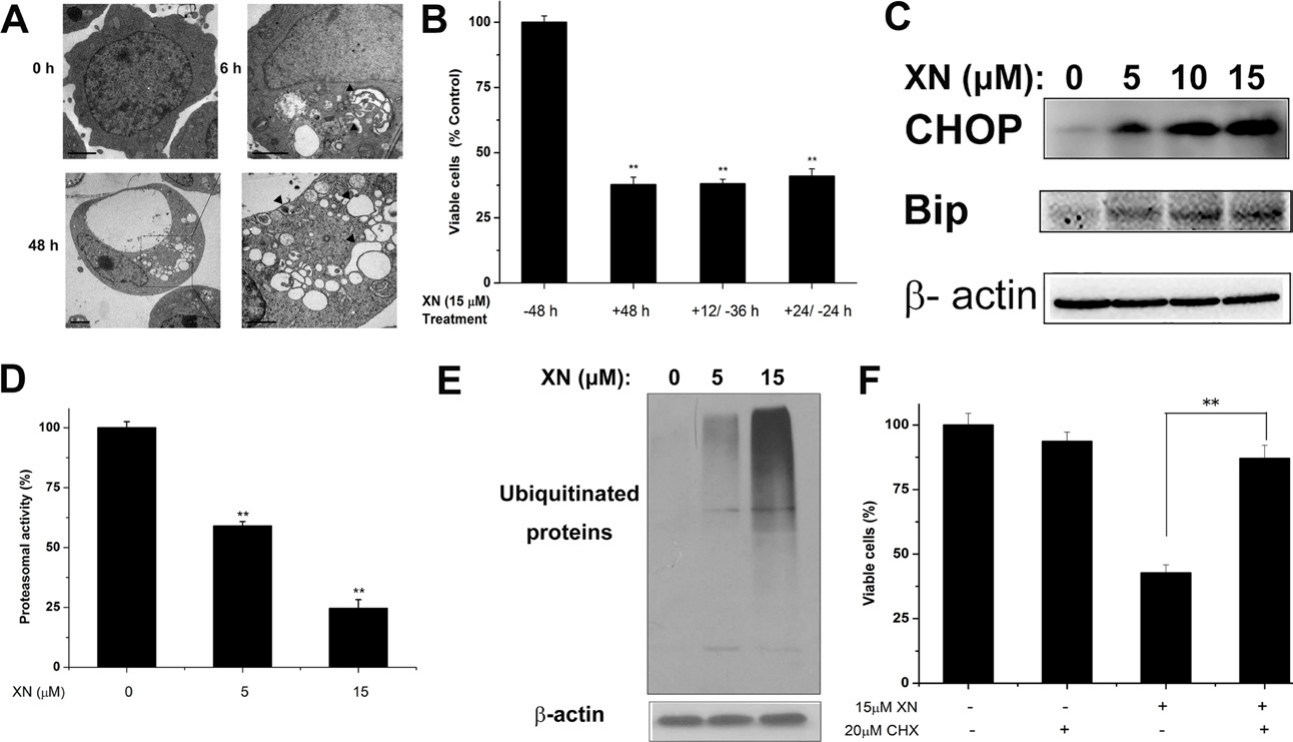
XN induces paraptosis of HL-60 leukemia cells (**A**) The cells were treated with 15 μM XN for the indicated time points and observed by transmission electron microscopy. Arrows indicate the normal ER in control cells and arrowheads indicate dilation of ER. Bars, 2 μm. (**B**) Decreased cell viability of HL-60 cells with either continuous presence of drug for 48 h or treated with drug for 12 h or 24 h and shifted to normal medium for remaining periods (+12/−36 h,+24/−24 h). (**C**) Western blot was used to determine protein levels of CHOP and Bip in cells treated with 5 μM, 10 μM, 15 μM XN for 48 h. (**D** and **E**) The cells were treated with 5 μM, 15 μM XN for 48 h, followed by measuring inhibition of the proteasomal activity using proteasome activity assay kit and Western blotting analysis using specific antibodies to ubiquitin (D). (E) The cells were pretreated with CHX (20 μM), after which cells were treated with 15 μM XN for 48 h. The percentage of viable cells was measured. All data are expressed as mean ± S.D. from three independent experiments. *P < 0.01 *vs*. control group.

XN treatment caused paraptosis of vacuolation without induction of caspase activation and autophagic flux (Figures 2 and 3). Because cytoplasmic vacuolation have been shown to be associated with paraptosis with dilatation of endoplasmic reticulum that induces ER stress [12, 13]. Cytoplasmic vacuoles initiated forming after 6 h of XN treatment and peaked at about 48 h in HL-60 leukemia cells. The intracellular vacuoles were enlarged gradually and the nuclei of XN treated cells were located peripherally and the XN treatment did not trigger fragmentation or chromatin condensation of the nuclei in HL-60 cells. In order to characterize the process of vacuolar formation, a transmission electron microscopy (TEM) was used to analysis of HL-60 leukemia cells at 6 h or 48 h after treatment with 15 μM of XN. Compared to the untreated cells, cells incubated with XN for 6 h showed that cytoplasmic vacuoles look like be fused and the swollen ER cisternae occurred in the HL-60 cells. The leukemia cells were incubated with XN for 48h demonstrated one large and massive smaller cytoplasmic vacuoles. The vacuoles were lacked of visible cytoplasmic organelle, it indicated that XN treatment-induced cytoplasmic vacuoles were derived from the dilated endoplasmic reticulum (ER) cisternae in the HL-60 leukemia cells (Figure 4A). Cells were incubated with XN for 12 or 24 hours, then washed and maintained in XN-free medium for another 36 or 24 hours. Intriguingly, there were no vacuolated cells visible after removing XN and further incubated with XN-free culture medium, and the trypan blue assay was employed to analyze the viable cells after XN-treatment and removal of XN at different times (Figure 4B). The result suggested that the cytoplasmic vacuolation was reversible but it lead to irreversible cell injury. Then the protein expression levels of the ER stress markers, CHOP and Bip/Grp78 caused by XN treatment were determined in HL-60 leukemia cells. Treatment with 5 μM, 10 μM, and 15 μM XN for 48 h significantly increased the protein levels of CHOP and Bip/Grp78 in HL-60 leukemia cells (Figure 4C). Recently, other investigators reported that the proteasome dysfunction is required for the dilation of mitochondria/endoplasmic reticulum [14]. Thus, we asked whether XN treatment also impairs proteasome activity in HL-60 leukemia cells. We found that XN-treated cells significantly inhibited proteasome activity (Figure 4D) and accumulated poly-ubquitinated proteins (Figure 4E). Therefore, the results indicate that XN-induced death of HL-60 leukemia cells under certain dose and time conditions was paraptosis. The paraptosis needs novo protein synthesis [15], then we further determined whether a new protein synthesis is involved in the XN induced-paraptosis. The HL-60 leukemia cells were administrated with the protein synthesis inhibitor, cycloheximide (CHX) that attenuated cell death in the XN treated HL-60 leukemia cells (Figure 4F).

### p38 mitogen activated protein kinase mediates paraptosis by XN

**Figure 5:**
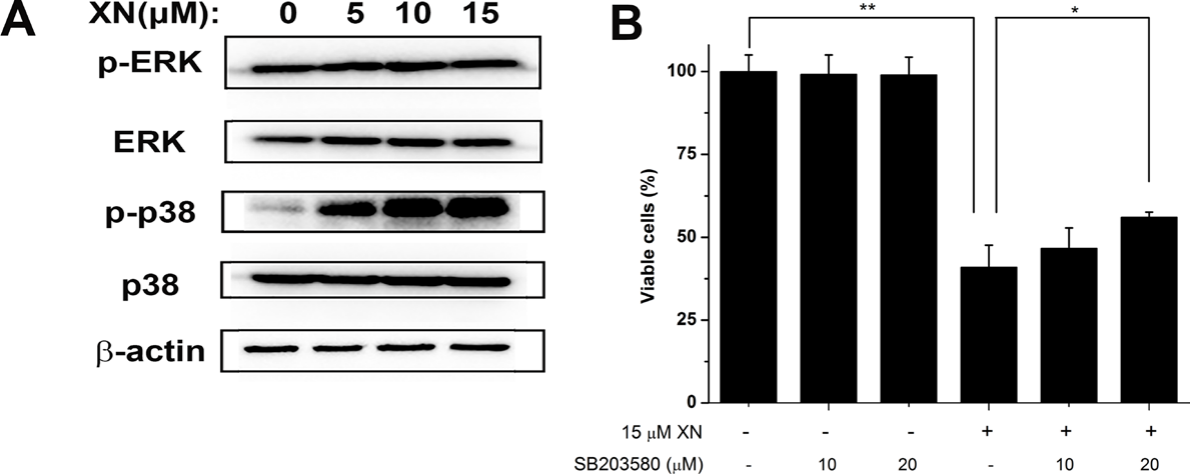
p38 mitogen activated protein kinase mediates XN-induced paraptosis (**A**) The HL-60 leukemia cells were treated with 5 μM, 10 μM, 15 μM XN for 48 h and the total and relative phosphorylation levels of the mitogen activated protein kinase (MAPK) were determined by Western blot. (**B**) The cells were untreated or pretreated with the indicated specific p38 inhibitor (SB203580) at the indicated concentrations for 1 h and further treated with 15 μM XN for 48 h. The cellular viability was assessed using trypan blue staining. All data are expressed as mean ± S.D. from three independent experiments. *P < 0.05, **P < 0.01 *vs*. control group.

In order to elucidate the signaling mechanisms by which XN treatment induced paraptosis in the HL-60 leukemia cells, we monitored the phosphorylation of p38 mitogen activated protein kinase (MAPK) by immunoblotting. The immunoblotting results showed that p38 MAPK was progressively activated but extracellular signal-regulated kinase (ERK) did not alter (Figure 5A). The importance of these MAPK signals were characterized by their specific inhibitors respectively, we found that XN-induced cell death of HL-60 leukemia cells was significantly inhibited by p38 MAPK inhibitor SB203580 (Figure 5B). Overall, these results demonstrated that XN-induced cell death of HL-60 leukemia cells is paraptosis that was characterized by cytoplasmic vacuolation without caspase activation and autophagic influx. The dilated endoplasmic reticulum and up-regulation of ER stress markers in HL-60 leukemia were also the characteristics of XN-induced cell death. The p38 MAPK triggered by XN treatment may play a crucial role in the XN induced-paraptosis.

## DISCUSSION

The elimination of malignant cancer cells usually depends on apoptotic pathways. There are more evidence noted that some of non-apoptotic pathways may contribute to tumor cell death induced by anti-cancer drugs. The caspase activation plays a critical role in apoptosis and the apoptosis is characterized with morphological and biochemical features such as chromatin fragmentation and alterations in apoptotic proteins [3, 16]. The paraptosis is not apoptotic cell death and the physiological roles and molecular pathways that involved in the paraptosis are not completely understood [17]. There are more evidence that as compared to normal cells, tumor cells usually demonstrate higher levels of endoplasmic reticulum (ER) stress [18, 19]. Thus, induction of paraptosis that targets mitochondria/endoplasmic reticulum (ER), could offer an attractive approach to kill cancer cells with resistance to the conventional pro-apoptotic chemotherapies.

The mechanisms by which XN exerts its anticancer activity include the chemo-preventive activity through attenuation of the development of carcinogenesis [20] reduction of proliferation, apoptosis inhibition [21] and block of migration [22], attenuation of angiogenesis [23] and autophagy [24]. Our current data for the first time demonstrate that XN could kill cancer cells via induction of paraptosis. The activation of caspase in the HL-60 leukemia cells by XN treatment was examined, the activation of caspase 3 and the cleaved fragments of caspase-3 were not detected, indicating that caspase activation is not involved in XN-induced paraptosis of HL-60 leukemia cells. The results from the caspase inhibitors also verified that there is no involvement of caspase activation in XN treated HL-60 leukemia cells. Because XN treatment resulted in cytoplasmic vacuolation in the HL-60 leukemia cells, we determine whether autophagic flux was involved in the XN-treated HL-60 leukemia cells. The results demonstrated that XN treatment up-regulated the levels of LC3-II and p62 but did not alter Beclin-1 levels. The pretreatment of HL-60 leukemia cells with the inhibitor of autophagic flux did not observe the alterations in the numbers of viable cells. It indicated that autophagy was inhibited at the fusion of autophagosomes with lysosomes step and not involved in the XN induced cell death.

XN treatment induces swelling and fusion of the endoplasmic reticulum, and results in the formation of expanded endoplasmic reticulum structures. In terms of these interesting finding, the signaling pathways responsible for the dilation of the endoplasmic reticulum (ER) during XN-induced paraptosis of HL-60 leukemia cells were determined. The unfolded proteins accumulation can activate the intracellular signaling pathways that trigger the unfolded protein response and endoplasmic reticulum-associated degradation to prevent the cells from toxicity of unfolded and mal-folded proteins. In this regard, we found that XN treatment significantly inhibited the proteasome activity and induced generation of unfolded proteins and the ER-stress markers such as transcription factor CHOP and Bip/Grp78 and thus may lead to cell death. Furthermore, the CHX pretreatment attenuated the XN-induced vacuolation and paraptosis. The results suggest that a protein synthesis is required for XN treatment induced dilation of endoplasmic reticulum of HL-60 leukemia cells. When we examined the activity changes in the mitogen-activated protein kinases, we found that p38 but not ERK was activated. The pretreatment with only inhibitor of p38 but not ERK, the cell survival rate were significantly rescued. Therefore, p38 activation was shown to be critical for paraptosis induced by XN.

In conclusion, we have reported for the first time that XN treatment can induce paraptosis of HL-60 leukemia cells. The paraptosis is characterized by cytoplasmic vacuolation without caspase activation and autophagic flux. The dilatation of ER and the accumulation of unfolded proteins and ER stress marker proteins were also observed in XN-treated HL-60 leukemia cells. Moreover, p38 MAPK specific inhibitor significantly prevented XN-induced paraptosis indicating the involvement of p38 pathways in XN-induced paratosis. Therefore, XN induced paraptosis could be a potential implication on cancer therapeutics.

## MATERIALS AND METHODS

### Chemicals and antibodies

Xanthohumol was obtained from Yumen Technology Development Limited Company (Gansu, China) with a purity ≥ 98% determined by HPLC. Xanthohumol stock solutions were prepared in dimethyl sulfoxide (DMSO) and diluted to the indicated concentrations before use. Trypan blue was purchased from Sigma-Aldrich (USA). The Cell Counting Kit-8 assay was obtained from Dojindo Laboratories (Japan). Caspase inhibitors Z-VAD-FMK was purchased from Medchemexpress (USA). Cycloheximide (CHX), SB203580 and 3-methyladenine (3-MA) were purchased from Selleck (USA). The Cyto-ID^®^ green dye autophagy detection kit was from Enzo Life Sciences (Switzerland). The Proteasome Activity Assay Kit was purchased from Abcam (England). The following antibodies were used: anti-β-actin, anti-caspase-3, anti-CHOP, anti-p62 (Cell Signaling); anti-LC3B (Sigma-Aldrich); anti-Ubiquitin (Abcam); anti-phospho-ERK1/2, total ERK1/2, phospho-p38, and total p38 (Santa Cruz Biotechnologies); goat anti-rabbit IgG-HRP and goat anti-mouse IgG-HRP (Santa Cruz Biotechnologies).

### Cell culture and treatments

HL-60 cell line (human promyelocytic leukemia cell line) and SH-SY5Y cell line (human dopaminergic neuroblastoma cells) were obtained from the Shanghai Cell Bank. HL-60 leukemia cells was propagated in Iscove's modified Dulbecco's medium (IMDM) supplemented with 20% (v/v) fetal bovine serum, 2 mM L-glutamine, 100 IU/mL penicillin and 100 μg/mL streptomycin at 37°C in air with 5% CO_2_ in a humidified incubator. The cells were passaged twice a week and used in the exponential growth phase. To assess viability, the cells were seeded (1 × 10^5^ cells/mL) in flat-bottomed 6-well tissue culture plates and incubated with different concentrations of XN for 48 h. SH-SY5Y cells were grown in Dulbecco's modified Eagle medium (DMEM) supplemented with 10% fetal bovine serum, 2 mM L-glutamine, 100 IU/mL penicillin and 100 μg/mL streptomycin at 37°C in air with 5% CO_2_ in a humidified incubator.

### CCK-8 assay

Cell viability was estimated by the CCK-8 assay. Approximately 10^4^ cells were seeded in 96-well plates with 100 μL medium each well. After 24 h cultivation, different doses of XN were added. Each well was incubated with 10 μg CCK-8 solution for 4 h away from light before measuring the absorbance at 450 nm by Multi-Mode Detection platform (Molecular Devices).

### Confocal microscopy

To visualize autophagic vacuoles and monitor autophagic flux in HL-60 leukemia cells with or without XN treatment, confocal microscopy was used. HL-60 leukemia cells were treated with 15 μM of XN for 48 h, with cells treated with 50 nM autophagy inducer rapamycin for 8 h as positive controls. Thereafter, cells were washed twice with culture medium and then treated with nuclear dye DAPI and autophagy detection kit Cyto-ID^®^ green dye at 37°C for 15 min. Confocal images were acquired using a Zeiss LSM-700 confocal microscope equipped with Plan-Apo 63 × 1.4 NA oil-immersion objectives. Fluorescence intensity was quantitatively analyzed using ZEN 2010 software (Carl Zeiss). Images were edited with Photoshop (Adobe).

### Transmission electron microscopy

After XN-treatment, cells were fixed in a fixative solution (2% formaldehyde and 2% glutaraldehyde in 0.1 M sodium cacodylate buffer, pH 7.4) at 4°C overnight. Then the cells were washed with 0.1 M sodium cacodylate buffer (pH 7.4) and post-fixed in 1% OsO_4_ for 1 h. Ultrathin sections were stained with uranyl acetate and lead citrate, and examined by transmission electron microscopy (JEM 1200 EX II, JEOL Ltd., Japan).

### Annexin V-FITC/PI assay

Apoptosis was examined using Annexin V Apoptosis Detection Kit FITC (Affymetrix eBioscience, USA). The HL-60 leukemia cells were prepared according to the manufacuturer's instructions. Briefly, approximately 1 × 10^5^ cells per experimental condition were harvested, washed with cold PBS twice, and resuspended with 200 μL Binding buffer. Adding 5 μL of fluorochrome-conjugated Annexin V solution and 10 μL PI solution, cells were incubated for 15 min at room temperature in the dark. Samples were analyzed by flow cytometry (BD). Data were analyzed using MODFIT software.

### Proteasome activity assay

Proteasome activity was measured by proteasome activity assay kit (Abcam) using a multi-mode detection platform. Fluorescence was measured using a Multi-Mode Detection platform (Molecular Devices) in the presence/absence of MG132 after 5 min at 37°C for 30 min. The MG132-sensitive increase of fluorescence at 350/440 nm was considered as proteasomal activity.

### Western blotting analysis

After XN-treatment, cells were lysed in RIPA buffer (Beyotime, Nanjing, China) and centrifuged at 14,000 × g for 20 min. Protein samples were separated electrophoretically by SDS-PAGE and transferred onto a polyvinylidene difluoride membrane. The membrane was incubated with 5% non-fat milk in TBST (Tris-buffered saline tween 20) at room temperature for 1 h. Washing with TBST, the membrane was incubated with primary antibody at 4°C overnight, and then incubated with horseradish peroxidas-conjugated secondary antibody at room temperature for 1 hour. The bound antibody was detected by chemiluminescence kit (Thermo scientific, USA) and immunoreactive protein bands were performed by densitometric analysis.

### Statistical analysis

Data are presented as means ± SEM from at least three independent experiments and evaluated by analysis of variance (ANOVA) followed by Student Newman-Keuls test. Values of *P* < 0.05 were considered statistically significant. All analyses were performed using the SPSS version 19.0.

## Abbreviations

AML: acute myeloid leukemia
Bip/GRP78: immunoglobulin heavy chain binding protein in pre-B cells
CHX: cycloheximide
CHOP: C/EBP homologous protein
GFP: green fluorescent protein
ER: endoplasmic reticulum
ERAD: ER- associated degradation
ERK: extracellular signal-regulated kinase
LC3: microtubule-associated protein 1 light chain 3
3-MA: 3-methyladenine
MAPK: mitogen activated protein kinase
SQSTM1/p62: equestosome 1
UPR: unfolded protein response
XN: Xanthohumol
